# Persistent susceptibility of *Aedes aegypti* to eugenol

**DOI:** 10.1038/s41598-022-06302-8

**Published:** 2022-02-10

**Authors:** Kamal Adhikari, Bulbuli Khanikor, Riju Sarma

**Affiliations:** Department of Zoology, Gauhati University, Guwahati, Assam India

**Keywords:** Biochemistry, Biotechnology, Drug discovery, Zoology, Environmental sciences, Health care

## Abstract

Botanical insecticides are preferred for their environment and user-friendly nature. Eugenol is a plant-based monoterpene having multifarious biocidal activities. To understand whether eugenol would persistently work against *Aedes aegypti*, we performed larvicidal bioassays on thirty successive generations and determined median lethal concentration (LC50) on each generation. Results showed no apparent differences between LC50 at F0 (63.48 ppm) and F30 (64.50 ppm) indicating no alteration of susceptibility toward eugenol. To analyze, if eugenol has any effect on metabolic detoxification-associated enzymes, we measured esterases (alpha and beta), cytochrome P450, and GST activities from the survived larvae exposed to LC50 concentration from F0–F30. Results revealed a decrease of esterases, GST, and cytochrome P450 activities at the initial 4–8 generations and then a gradual increase as the generations progressed. GST activity remained significantly below the control groups. Synergists (TPP, DEM, and PBO) were applied along with eugenol at F30 and LC50 concentration, and the said enzyme activities were recorded. Results showed a noticeable decrease in LC50 and enzyme activities indicating effective inhibitions of the respective enzymes. Overall, present results inferred that eugenol would effectively work as a larvicide for a longer period in successive generations without initiating rapid resistance and therefore could be advocated for controlling *A. aegypti*.

## Introduction

*Aedes aegypti* bears immense epidemiological importance as the females of this mosquito are the vector of dengue, Zika, yellow fever, and chikungunya. For dengue alone, billions of people are at risk of contracting, millions of people are infected, and thousands of people are losing their lives annually. No vaccines or specific treatments are available to date against dengue, Zika, and chikungunya diseases. Killing the vector mosquitoes at their larval stages is the best effective measure to reduce the population densities of adult mosquitoes, which impacts the transmission of the diseases. Synthetic pesticides are the most widely used and relied upon method of mosquito control. Initially, DDT (an organochlorine pesticide) was used to control mosquitoes. However, due to resistance development by mosquitoes and environmental concerns, DDT was replaced by other classes of pesticides such as carbamates, organophosphates, and pyrethroid^1^. Moreover, the extensive and long-term use of these insecticides has led to the accumulation of their residues in food, cow milk, water, soil, as well as other environmental components. There are also reports of hormonal alterations in persons engaged in spraying and using these insecticides^2^.

Long-term studies with the organophosphates malathion and temephos and with the pyrethroid deltamethrin reported resistance development in insect pests^3–5^. Therefore, as an alternative, plant secondary metabolites (essential oils, extracts, and their phytochemical components) are increasingly being claimed to be effective as promising insecticides^6^. Among these metabolites, terpene compounds are extensively studied owing to their broad-spectrum applications^7^. All terpene compounds have a common precursor- isopentyl pyrophosphate. These compounds can be categorized into several groups based on the number of carbon atoms present; hemiterpene (5C), monoterpene (C10), sesquiterpene (C15), diterpene (C20), sesterpene (C25), triterpene (C30), tetraterpene (C40) and polyterpenes ( C40 and above carbon units)^8^. Terpene compounds exert their toxic effects in several ways. The principal pathways include the cholinergic system^9^, GABA system^10^, mitochondrial system^11^, and the octopaminergic system^12^. It can disturb the endocrinological balance and serves as an insect growth regulator^13^. It also affects respiration by blocking the spiracle of the larvae restricting their ability to breathe^14^.

Here in the study, we selected eugenol, a monoterpene, the main constituent of basil and clove essential oil. It has been proven effective in controlling a wide range of pests, including mosquitoes^15–17^. Apart from that, eugenol has also been proven effective in numerous human applications such as dentistry^18^, anticancer^19^, antimicrobial^20^, and antioxidant^21^. From the point of efficacy and user acceptability, eugenol is an attractive compound to be used as a mosquitocidal agent. However, with the introduction of a novel pesticide, there is always a possibility of resistance development by the target pest; thus, its long-term efficacy must be evaluated. Resistance is a complex phenomenon in which the recommended dose of insecticide is not able to kill the majority of insect populations at subsequent application. Insects can develop resistance through target-site insensitivity, metabolic resistance, and reduced cuticular penetration^22^. Among these mechanisms, metabolic resistance mediated by the complex multigene enzyme family of esterase, glutathione-s-transferase, and cytochrome P450 is prominent and well established^23–25^. Therefore, to evaluate the persistent toxicity of eugenol, the study was performed to determine LC50 concentration in each generation, from F0 to F30, and to spectrophotometrically estimate the said enzymes, using suitable substrates, to examine the effect of eugenol on these detoxifying enzymes.

Synergists like triphenylphosphate (TPP), diethyl maleate (DEM), and piperonyl butoxide (PBO) could inhibit esterases, glutathione-s-transferase, and cytochrome P450s, respectively. This inhibition enhances the toxicity of insecticides^26–28^. Such studies establish the importance of metabolic detoxification-associated enzymes in resistance. Therefore, an attempt was made to assess the effect of the combination of synergists with eugenol on (1) the toxicity in terms of LC50 and (2) the detoxification-associated enzyme activities on F30 larvae of *A. aegypti*.

## Materials and methods

### Establishment and maintenance of *A. aegypti* colony

The rearing of mosquitoes was done following the method described by earlier authors^29–31^. Egg strips were kept submerged in a shallow rearing tray containing almost 2 L of water. After emergence, larvae were fed with a diet of dog biscuit (Pedigree) and yeast powder, in the ratio of 3:1. After 5/6 days of emergence the larvae metamorphosed into pupae. The pupae were collected in plastic cups holding almost 150 ml water and put in an adult rearing cage. Adults emerged from the pupae after 1–2 days of pupal period. Adults were fed 10% glucose solution soaked in cotton. After 5 days of adult emergence, along with sugar solution, the adult females were offered blood meals from albino rats to obtain progeny. Each rat was used only once a week. For egg-laying, filter paper submerged in water in a glass beaker was provided. The culture was maintained at 28 ± 2˚C and 75 ± 5% RH, at 12:12 light: dark cycle. Ethics approval: the Institutional Animal Ethics Committee (IAEC), Gauhati University, had approved the protocol for the study (Permit No. IAEC/Per/2020-21/08). The guidelines for laboratory animals prescribed by the Committee for the Purpose of Control and Supervision of Experiments on Animals (CPCSEA), a statutory committee under the Ministry of Environment and Forests (Animal Welfare Division), Government of India, were strictly followed while using rats for the experiment. For the blood-feeding purpose, we followed the method as described by Morlan et al^31^ for *A. aegypti* and the guidelines formulated by the Ethiopian Public Health Institute (EPHI) for mosquito rearing (Anopheles) and insectary handling^32^ with few modifications.

Initially, the eggs were obtained from the Regional Medical Research Center (RMRC), Dibrugarh, Assam, India. In RCMR, the mosquito colony was established from field-collected *A. aegypti*, and reared for almost 14 years without exposure to any form of insecticides. In our laboratory (Laboratory of Entomology, Gauhati University), the mosquito colony was reared for about six years with no exposure to insecticides. So, by the time the bioassays were carried out, the mosquitoes were 20 years of culture in the laboratory.

### Larvicidal activities

The larvicidal bioassay was carried out following the method of the world health organization (WHO)^33^. Graded concentrations of eugenol- 1, 5, 10, 25, 50, 100, 250, and 500 ppm were prepared using DMSO as an emulsifier. Four replicates were prepared for each concentration of eugenol in 100 ml water. In each replica 25 number of 4th instar larvae were introduced. An equal number of negative and no-treatment controls were also set using DMSO-water and water only, respectively. The mortality of the larvae was constantly monitored, and data was recorded at 5 min, 10 min, 15 min, 30 min, 1 h, 2 h, 3 h, 4 h, 5 h, 6 h, and 24 h, respectively. Experiments were set in a separate laboratory away from the mosquito culture room. If any of the 4th instar larvae pupated during the exposure period, it was negated from the test, again, if more than 10% larvae from the control group died, the whole experiment was repeated. Larvae that did not show movement even after touching with a fine brush were considered dead. If mortality of control larvae occurred below 10%, the mortality of the treated groups was calculated using Abbott’s formula^34^.

$${\text{Mortality }}\left( \% \right) \, = \left( {{\text{C}} - {\text{T}}} \right)/{\text{C}} \times {1}00$$

where C = percentage of larvae survived in the control group, T = percentage of larvae survived in the treated group.

Mortality percentage was calculated after 24 h exposure period.

From the same larvicidal experiment, median lethal concentration (LC50) was calculated. The mortality values were analyzed through SPSS software using Probit analysis^35^. Log concentrations and probit obtained from the SPSS software (version 20) were then analyzed using MINITAB software. This LC50 was considered the LC50 for F0 generation.

### Selection of larvae from F0 to F30 generation

From the original susceptible colony of mosquitoes, approximately 3000 fourth instar larvae were separated and introduced to a median lethal concentration (LC50) of eugenol determined as stated above for 24 h. Survived larvae were separated and transferred to clean water, and food was provided. When these larvae pupated, they were allowed to metamorphose into adults. The progeny obtained from those adults was the F1 generation. When the F1 larvae grew to the fourth instar, they were again used for the determination of LC50 using the same series of concentrations as described for F0. The newly calculated LC50 was the LC50 for F1. This LC50 was applied to approximately 3000 F1 larvae for 24 h. The survived larvae were separated, food was provided, and allowed to continue the next generation. The same procedure was followed for F2–F30 generations.

Simultaneously, metabolic detoxification-associated enzyme activity was determined using some of the larvae that survived the F0 selection. Likewise, the enzyme activity was determined using some of the F1 larvae that survived the F1 selection. The same procedure was followed for determining the enzyme activity of the remaining F2–F30 generations.

### Estimation of the detoxification enzyme activity

Three principal detoxification enzymes involved in metabolic resistance, viz. esterases, GST, and cytochrome P450 monooxygenase were quantified following the method as described by Safi et al^36^.

### Sample

Homogenate was prepared from larvae that survived the selection pressure. In each replica, three larvae were taken and homogenized in 900 µl (300 µl for each larva) 0.0625 M potassium phosphate buffer (pH 7.2) in a 1.5 ml microtube. Four replicates for each treatment and an equal number of controls were set. The larvae exposed to eugenol were used as treatment group, exposed to emulsifier DMSO were used as a negative control group whereas the larvae in tap water was used as a no-treatment control group. The samples were cold centrifuged at 10,000 rpm for 10 min at a controlled temperature of 4ºC, and the supernatant obtained was used as a crude enzyme extract for spectrophotometric determination of enzyme activities.

### Esterase

Alpha naphthyl/beta naphthyl acetate was used as a substrate for the quantification of alpha and beta esterase, respectively. 200 µl of 30 mM of (alpha/beta) naphthyl acetate was mixed with 100 µl insect homogenate. Allowing to stand for 30 min, 10 µl 0.3% fast blue stain was added. Blank was also prepared in a similar manner in distilled water instead of insect homogenate. After 5 min, OD was measured at 570 nm. The mean of ODs was converted to product concentrations using the standard curve of alpha/beta naphthol. Enzyme activities are presented as μM of product formed/min/mg protein.

### Glutathione-s-transferase

GST activity was quantified using a mixture of 200 μl of 10 mM GSH and 3 mM 1-chloro-2,4-dinitrobenzene (CDNB) (the mixture of these two chemicals is referred to as cocktail). 10 µl of insect homogenate was taken to which 100 µl of cocktail buffer was added. After 5 min, OD was taken kinetically for 5 min at an interval of 1 min. GST activity was quantified as mM of conjugate produced/min/mg protein taking the extinction co-efficient of CDNB multiplied by the path length.

### Monoxygenase

3, 3, 5, 5-Tetramethyl benzidine (TMBZ) was used as a substrate for quantifying P450 activity. To a 20 µl of larval homogenate, 80 μl of 0.625 M potassium phosphate buffer (pH 7.2), 200 µl TMBZ (0.01 g TMBZ in 5 mL methanol + 15 mL sodium acetate buffer pH 5.0), and 25 μl 3% hydrogen peroxide were added. The mixture was incubated at room temperature for 2 h, and OD was measured at 450 nm. Standard concentrations (0.01, 0.02, 0.04, 0.06, 0.08, and 0.1 mg/mL) of cytochrome C were estimated and plotted as a graph in MS-Excel. From the standard graph, the unknown concentration of P450 monooxygenase was calculated and expressed as cytochrome P450/min/mg protein.

### Protein assay

Protein estimation was done following the method of Lowry et al^37^, taking BSA as standard. Samples were prepared in quadruplet and OD was measured at 660 nm.

### Efficacy of synergist

The best-known synergists- PBO, TPP, and DEM, known to inhibit cytochrome P450, esterase, and GST, respectively, were bought from MERCK and used with eugenol. For the synergistic experiment, application method as described by Xu et al^4^ was followed, with some modifications. While for the larvicidal bioassay, the method of WHO^33^ was followed as described above. For each liter of water, 5 mg of synergists (PBO, TPP, and DEM) was used. The synergized water was used to prepare a graded concentration of eugenol (1, 5, 10, 25, 50, 100, 250, and 500 ppm), and 25 numbers of F30 larvae were introduced into each concentration. Larval mortality was monitored at different time intervals, and median lethal concentration was calculated at 24 h. The LC50 thus calculated was applied to a large mass of larvae in one liter of synergized water for 24 h. From the survived larvae, enzyme activity was determined after 24 h.

### Approval for animal experiments

The protocol for the study was approved by the Institutional Animal Ethics Committee (IAEC), Gauhati University (Permit No. IAEC/Per/2017/RF/2018-05).

## Results

**Figure 1:**
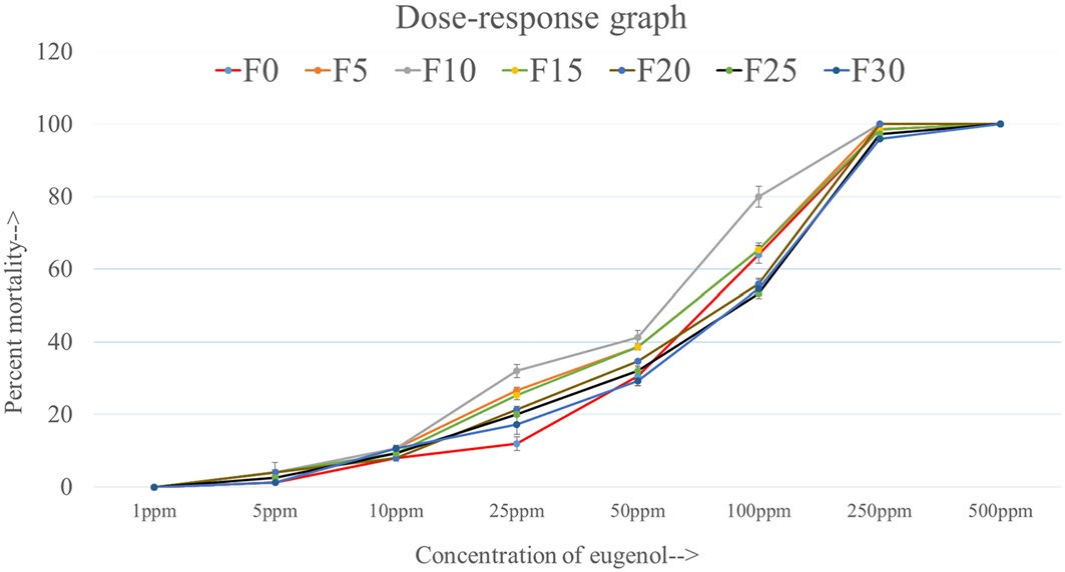
Response of 4th instar larvae to a graded concentrations of eugenol over a generational time (± SE).

Figure 1 represents the percent mortality of larvae in response to increasing concentrations of eugenol. As evident, eugenol exhibited dose–response mortality in larvae and did not show a noticeable decreasing or increasing effect on mortality over 30 generations. When plotted for every 5th generation, it appeared nearly overlapping graphs. Three of the tested concentrations—25 ppm, 50 ppm, and 100 ppm showed a modest difference in mortality percent, whereas the rest of the concentrations 1 ppm, 5 ppm, 10 ppm, 250 ppm, and 500 ppm exhibited almost similar larval mortality over a generational time (Fig. 1 and Supplementary Table 1).

### LC50 values of the larvae from F0 to F30 generations

**Figure 2:**
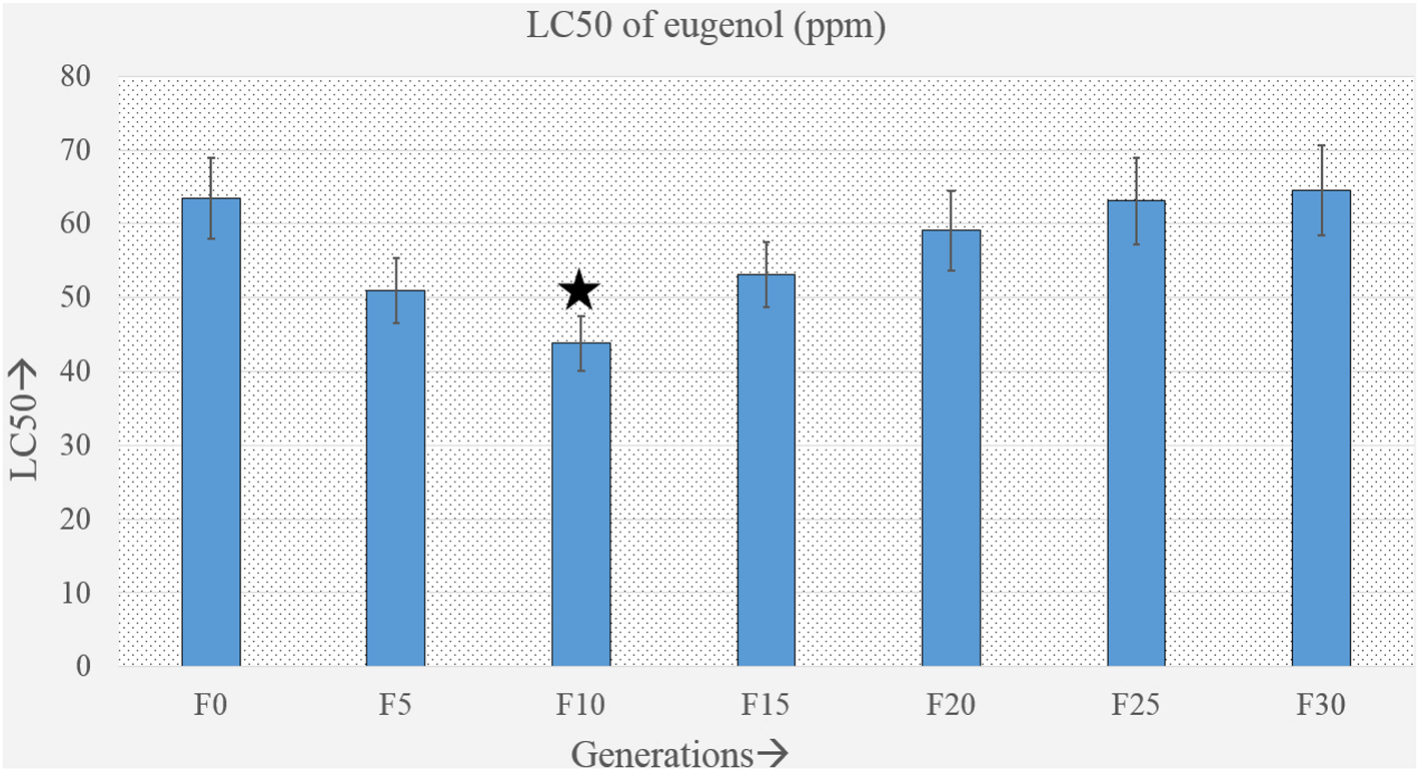
Bar diagram showing the median lethal concentration of eugenol on every fifth successive generations of *A. aegypti* upto F30 generations. 95% confidence intervals for each LC50 are presented in the form of error bars.

The bar diagram (Fig. 2) of the median lethal concentration (LC50) shows the initial decrease in the LC50 value, which was significant between F0 and F10, and then a gradual increase, with the increasing trend which continued up to the last generation studied. No significant difference in LC50 was found between F0 and F30 but was found to be significant between F0 and F10. The LC50 value dropped from 63.48 ppm in the F0 to 43.79 ppm in the F10 before progressively rising following the F10 generation (Supplementary Table 2). It remained fairly constant from F24 to F30 generation (63–65 ppm), which was almost similar to F0. Although there were fluctuations between generations, over 30 generations of exposure, the mosquitos showed no obvious changes in susceptibility to eugenol in terms of median lethal concentration. Rather, susceptibility increased up to the first ten generations as indicated by the low LC50 value. However, the mosquito began to adapt in the later generations, followed by a rise in LC50, and by the F30 generation, there was no apparent difference in susceptibility between F0 and F30 (Supplementary Table 2).

### Quantification of enzyme activity

#### Esterases

**Figure 3:**
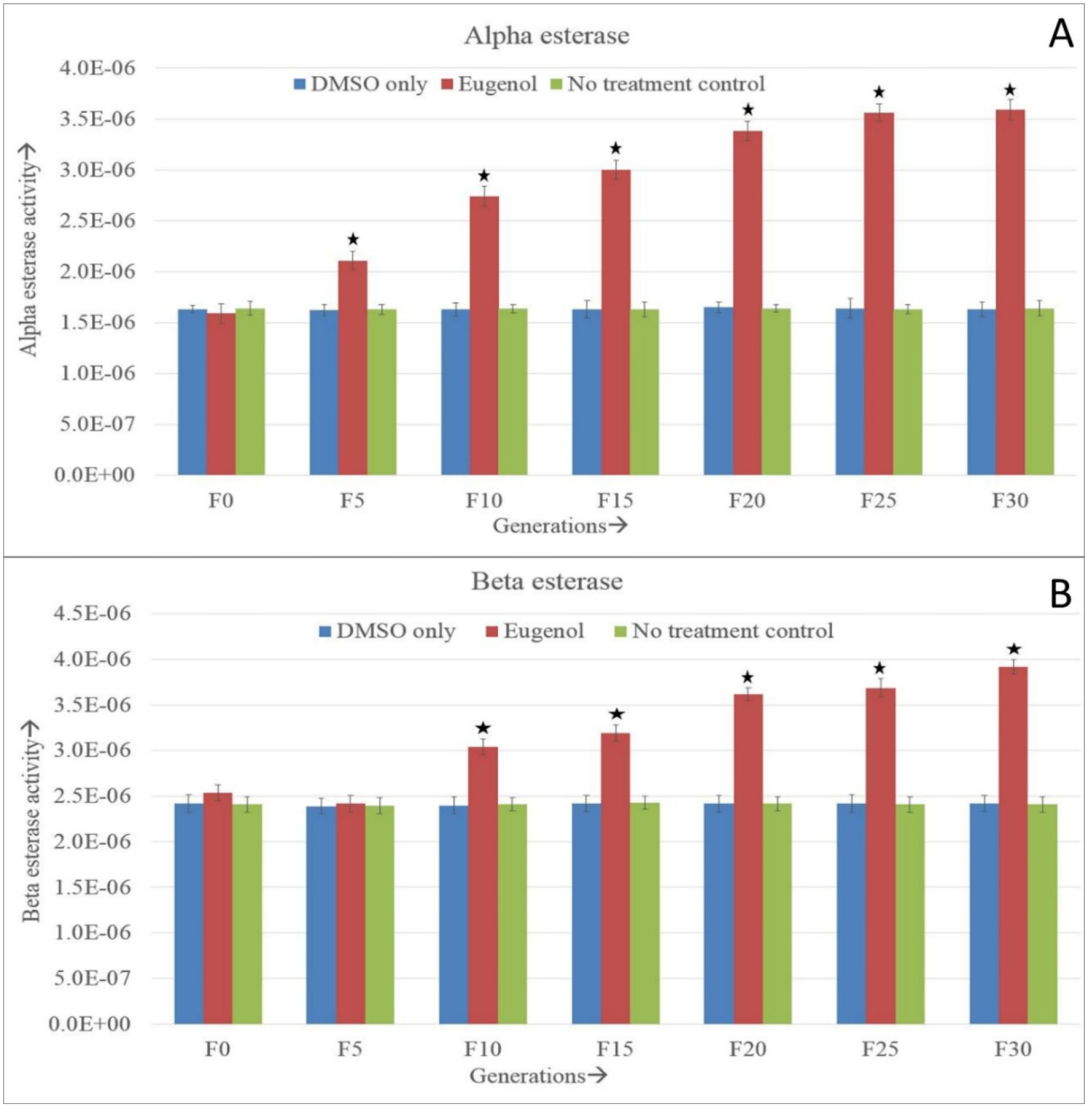
(**A**) α-esterase enzyme activity (± SE), (**B**) β-esterase enzyme activity (± SE) in continuously exposed (F0 to F30) *A. aegypti* larvae. The asterisks represents the significant difference between the experimental groups. Tukey’s post hoc test was employed to determine the significance in differences. The enzyme activity is expressed at the unit of μM of product formed/min/mg protein. *The mean difference is significant at the 0.05 level.

Alpha esterase activity was found almost constant at F0, F1, and F2 generation of exposure. The enzyme activity of treated larvae was found significantly different compared to that of the control group from the F3 till F30 generation. It rose above the control groups in the 5th generation, and the titer remained elevated in the subsequent generations. However, from F21 onwards, the enzyme activity became quite steady (Fig. 3A,B and Supplementary Table 3).Throughout the generations, the negative and no treatment control groups remained almost constant with no significant difference.

The Beta esterase enzyme activity of the treated group was inconsistent up to the F5 generation. In the F0, F1, F5 and F6 generations, it was found constant with the control groups. In the rest generations, it differed significantly. In the F7 generation, beta esterase activity rose significantly above the control group, and from the F7 to F30 generation, a gradual and significant rise in the treated group enzyme activity was observed (Fig. 3B and Supplementary Table 3). However, from the F21 generation onwards, the enzyme activity became almost steady. No significant difference between the negative and no-treatment control groups was found in any generations.

### Glutathione-s-transferase

**Figure 4:**
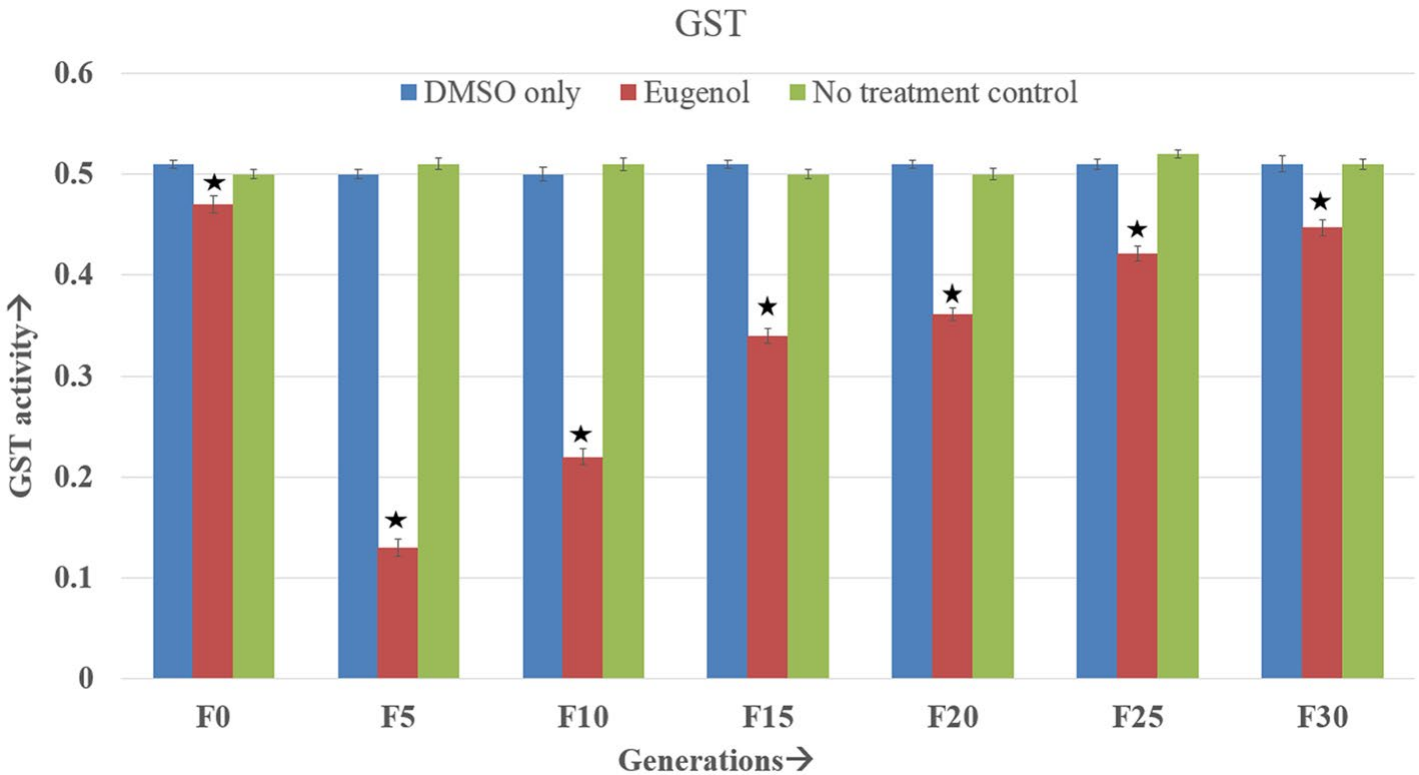
GST enzyme activity (± SE) in continuously exposed (F0 to F30) *A. aegypti* larvae. The asterisks represents the significant difference between the experimental groups. Tukey’s post hoc test was employed to determine the significance in differences. The enzyme activity is expressed at the unit of mM of conjugate produced/min/mg protein. *The mean difference is significant at the 0.05 level.

In all generations studied, the GST enzyme titer remained below the level of control groups. It began to decrease from the F0 generation itself. F5 generation experienced the lowest GST activity. With the passage of generations, though it began to rise gradually, it still remained significantly below the control groups (Fig. 4 and Supplementary Table 3). However, from the F27 generation, the enzyme activity became quite steady. No significant difference between the negative and no-treatment control groups was found.

### Cytochrome P450

**Figure 5:**
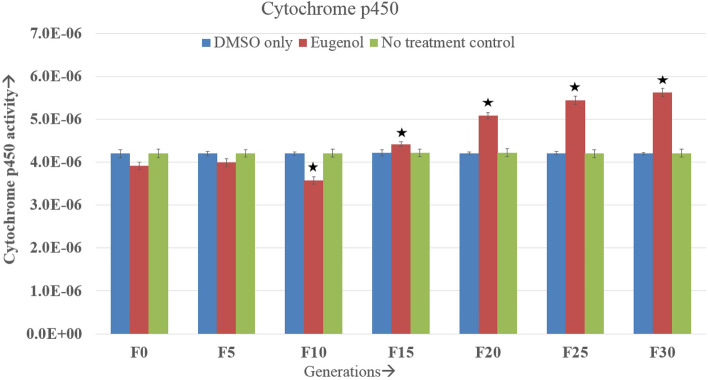
Cyt P450 enzyme activity (± SE) in continuously exposed (F0 to F30) *A. aegypti* larvae. The asterisks represents the significant difference between the experimental groups. Tukey’s post hoc test was employed to determine the significance in differences. The enzyme activity is expressed at the unit of cytochrome P450/min/mg protein. *The mean difference is significant at the 0.05 level.

Initially, the p450 enzyme activity decreased from the level of control groups and, after certain generations, it began to rise. During the F0 to F5 and again in the F13 and F14 generations, the treated and control enzyme activity were almost at par. During the F6–F12 generation, the treated enzyme titer decreased significantly compared to control groups, and from the F15 generation onwards, it began to rise significantly. This rise in enzyme titer continued till the last generation studied. The enzyme titer showed a regular trend of increase and decrease (Fig. 5 and Supplementary Table 3). However, after the passage of the F24 generation, it became almost steady. A significant difference between the negative and no-treatment control groups was not found.

### Efficacy of synergists

**Figure 6:**
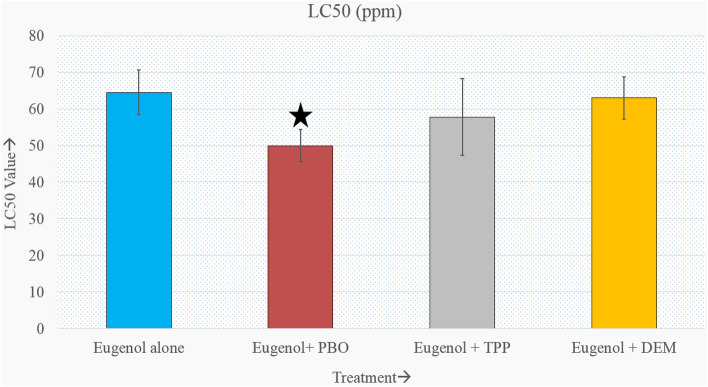
Effect of synergists + eugenol on the median lethal concentration (LC50) on the F30 larvae of *A. aegypti*. As the 95CIs overlap between all three synergized eugenols, we have compared eugenol + PBO and Eugenol alone. 95% confidence intervals for each LC50 are presented in the form of error bars for each synergized treatment.

While using eugenol in combination with PBO, TPP, and DEM specific synergist for P450, esterases, and GST, respectively, toxicity variations reflected in mortality in terms of LC50 value changes were observed (Fig. 6). While using PBO, the significantly higher toxicity of eugenol was observed, which was reflected by a decrease in LC50 value from 64.50 to 49.98 ppm. While using TPP, we recorded a non-significant higher toxicity of eugenol. Whereas DEM, a specific inhibitor of GST, was found to have vey negligible effect on increasing the toxicity of eugenol (Supplementary Table 4).

**Figure 7:**
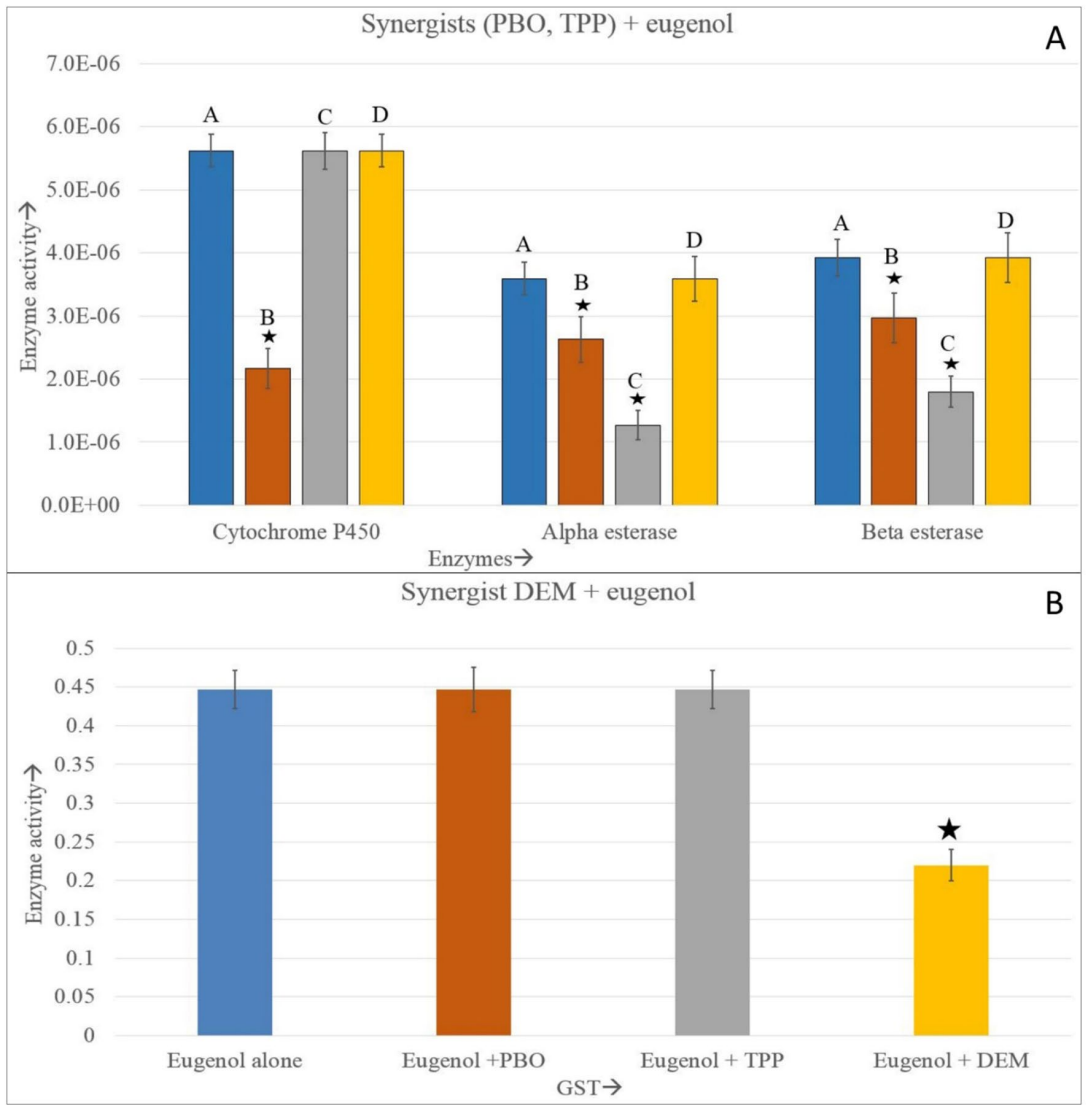
Effect of synergists (PBO, TPP and DEM) on the metabolic detoxification enzymes- esterases, GST and cytochrome P450 of *A. aegypti*. Asterisks represents the significance in difference and both directional vertical bar at the tip of each enzyme bar represents the error bars. Enzyme activity of esterases, cytochrome p450, and GST are expressed at the unit of μM of product formed/min/mg protein, cytochrome P450/min/mg protein, and mM of conjugate produced/min/mg protein, respectively.

In the Fig. 7A, A = eugenol alone, B = eugenol + PBO, C = eugenol + TPP, D = eugenol + DEM.

As is evident, the PBO + eugenol combination had an effect on cytochrome P450 as well as on esterase enzymes, as both of these enzymes decreased significantly. It had the highest effect on P450 enzymes because this enzyme activity decreased almost threefold. However, the combination had no effect on GST enzymes. The combination of TPP and eugenol significantly inhibited esterases but had no effect on GST and P450. Similarly, the combination of DEM and eugenol affected GST activity significantly but had no effect on esterases and cytochrome P450 (Fig. 7 and Supplementary Table 5).

## Discussion

Eugenol, a monoterpenoid commonly present in a major portion of clove, basil, nutmeg, and cinnamon essential oils, is a compound of interest in the medicinal, pharmaceutical, cosmetic, insecticidal, and food industries because of its multifarious properties. This phenolic terpene compound has been reported effective against a wide range of human ailments. It has been proved safe in food packaging^38^, antiseptics in the food industry^39^, food preservatives^40^. The environment protection agency (EPA) has approved eugenol use as an active insecticide ingredient because of its low mammalian toxicity^41^. In the research area of mosquito control, eugenol is reported to have mosquitocidal properties^42,43^, but a detailed investigation is wanting. So, the present investigation aimed to investigate the persistent susceptibility of *A. aegypti* toward eugenol, taking two primary parameters- toxicity in terms of median lethal concentration (LC50) and metabolic detoxification associated enzyme activities up to thirty successive generations.

As evident from the results, the median lethal concentrations (LC50) of eugenol against the larval stage of *A. aegypti* was below 65 ppm across the generations. While applying eight concentrations, dose-dependent enhancement of mortality was observed in all the cases. The higher concentrations (250 ppm and 500 ppm) resulted almost 100 percent mortality of all the treated larvae in all the generations.

While looking at the values of LC50 obtained from the experiment, we observed an initially gradually decreasing trend up to F9, followed by a gradually increasing trend up to F24 and then a steady level till F30 generation (Supplementary Table 2). Though there was a fluctuation between F0 to F30, the initial and final LC50 values remained almost the same, which might imply that the treated *A. aegypti* population remained susceptible to eugenol for up to thirty successive generations. Susceptibility assessment across generations was examined in *Culex quinquefasciatus*, *A. aegypti* and *Aedes albopictus* against malathion, permethrin, and temephos by Hamdan et al^3^, where they showed that the median lethal concentration determined in one generation was not median lethal to the subsequent generation. They recorded in *A. aegypti* after exposure for 32generations, increase of resistance ratio by 4.97, 64.2, and 51.0 folds to malathion, permethrin, and temephos, respectively. Similarly, Hidayati et al.^5^ reported lowered susceptibility status of *A. aegypti* when exposed to malathion for successive generations. After 45 generations of exposure, they recorded 52.7 folds increase in resistance ratio compared to F0. In contrast we have not observed marked changes of susceptibility status in terms of LC50 values up to F30 generations**.** Rather, at the tenth generation the treated mosquito populations was observed to be more susceptible with reduced LC50 value.

Esterases, glutathione-s-transferase, and cytochrome p450 protect insects against the lethal effect of insecticides by detoxifying them. Esterases cleave the carboxyl ester and phosphodiester bonds of insecticides, inactivating them and rendering the chemicals amenable to excretion^44^. Because these bonds do not exist in eugenol, esterase may not be directly involved in eugenol metabolism, but it may be involved in the metabolism of eugenol conjugates and metabolites. Fischer et al^45^ recorded conjugates and nine metabolites of eugenol in human subjects. In the experiment, we noticed a gradual fall in both alpha and beta esterase activities initially up to 4–5 generations and then a gradual rise in the following generations, which was significant than the control group. The initial decrease of these enzymes in post-treatment matches with the findings of Koodalingam et al^46^, where they recorded a decline in esterase activity after treatment of *A. aegypti* with extract of soapnut *Sapindus emarginatus*. The initial decrease in the activity of esterases might be due to sudden shock of selection pressure which required some time to adapt, and also may be due to involvement in the detoxification process. So, the freely available enzyme titer decreased. Again, the increase in esterase activity on subsequent treatment matches with the findings of Cao et al^47^, where they recorded 4.54 fold increase in esterase activity in *Aphis gossypii*. The increased expression of alpha and beta esterases might require for providing life support in a stressful environment. Esterase comprises 0.4% of the total protein in the insect body^48^, which may increase by 50% upon the application of selection pressure to account for about 3% of the total body protein in insects^49^. Cao et al^47^ reported a significant rise in esterase activity in *Aphis gossypii* after continuous exposure for ten generations with omathoate.

GST catalyzes the conjugation reaction, in which it conjugates glutathione to electrophilic substrates. Glutathione may also be added to eugenol itself or the metabolites of eugenol and helps detoxification and subsequent excretion. While studying GST in continuously exposed populations, we observed that GST activity significantly decreased in all the treated generations compared to control. GST activity might be inhibited by eugenol, as Rompelberg et al^50^ reported the inhibition of GST upon eugenol exposure in rats, mice, and humans. When these enzymes are blocked, they become unable to metabolize insecticides, thereby toxicity of insecticides persists. Similar decrease in GST activity in *Brontispa longissima* fed with myristicine treated coconut leaves was reported by Qin et al^51^. The result tallied with the findings of Muthusamy and Shivakumar^52^, where they reported significant inhibition of GST in *Amsacta albistriga* on treatment with cypermethrin. Muthusamy et al^53^ also reported the inhibition of GST in *Spodoptera litura* on treatment with chlorantraniliprole, which was found in line with the findings of present investigation. Rachokarn et al^54^ showed that leaf extracts of *Melia azedarach* and *Amaranthus viridis* strongly inhibited the detoxification enzymes of *Spodoptera exigua*. Apart from their role in detoxification, GSTs are involved in important life parameters such as stress physiology and intracellular transport in insects^48^. As a result of inhibition of this enzyme, the normal physiology of insects may be disturbed, perhaps leading to mortality due to oxidative stress. Experiment conducted by Tang et al^55^, showed that GSTs are affected heavily in *Micromelalopha troglodyta* and *Clostera anachoreta* when treated with plant allelochemicals.

Cytochrome p450 monooxygenase is involved in organophosphate and pyrethroid resistance. Elevated levels of these enzymes accounts for the increased breakdown of insecticide, which alters the susceptibility status of insects^56^. P450 is involved in the detoxification of exogenous compounds. In the present investigation, P450 enzyme activity decreased till F8 generation, following which it gradually increased. By the F15 generation, the activity of P450 significantly rose above the control groups, and this rising trend continued in the later generations. A similar reduction in P450 following initial treatment was also recorded by Bullangpoti et al^57^ on *Spodoptera frugiperda* when treated with the senescent leaf extract of *Jatropha gossypifolia*. This enzyme is also involved in a wide range of biological activities such as the metabolism of juvenile hormones, synthesis and degradation of ecdysteroids, as well as the metabolism of fatty acids and pheromones^28^. As a result, lower enzyme activity could be linked to greater toxicity, as seen during the initial exposure.

For the confirmation of the involvement of the tested enzymes in the detoxification process, three specific synergists (TPP, DEM and PBO) were used individually with eugenol. On using TPP with eugenol, alpha and beta esterase enzyme activity were decreased significantly, but there was no apparent changes in LC50 value. This result matches with the reports of Koou et al^58^, where they recorded no significant increase in mortality after treatment with synergists. While combining DEM with eugenol, we recorded a significant decrease in GST activity but no effect on the toxicity of eugenol. Inhibition of GST activity post-treatment with DEM is well-established^59^. However, in the present study, no apparent alteration in toxicity was observed between DEM + eugenol treated group and eugenol alone group (63 and 64.50 ppm, respectively). But while combining PBO with eugenol, we recorded a significant decrease of P450 and esterase enzyme activities. PBO application imparted the highest toxicity to eugenol, as evident from the LC50 values. The median lethal concentration value when treated with eugenol alone and eugenol + PBO was 64.50 and 49.98 ppm, respectively. Similar to our results, Tak et al^28^ reported the inhibition of cytochrome P450 as well as esterases by synergist PBO in *Trichplusia ni*.

Overall, the results represent that the LC50 value initially decreased till F9 generation before rising to meet the original LC50 value. The activity of metabolic detoxifying enzymes particularly cytochrome p450 and GST followed a similar pattern showing initial decrease and then the GST activity began to rise from the F5 generation, and cytochrome P450 from the F8 generation onwards. Thus, we observed that there exists an association between enzyme activity and susceptibility of the larvae toward eugenol. During the initial treatment, all the tested enzymes produced might have been used in physiological processes and in the detoxification of eugenol into its less harmful products, resulting in a drop in enzyme activity. The enzymes produced might not have been sufficient to detoxify eugenol, which resulted in increased toxicity. Under continuous stressed conditions, detoxification enzymes tend to increase in concentration than their normal counterpart. This increased production might affect the fat bodies, the site for ecdysone synthesis, and also the main source of acetyl groups needed for the synthesis of constitutive amino acids as well as other vital body processes. It is probable that in the later generations with the activation of detoxifying genes, enzymes were produced in larger quantities that led to increased detoxification and a subsequent rise in LC50. Though the exact mechanism of stimulation or decline of enzyme activity is not clear, synthesis of new protein after an exogenous compound binds to a cytosolic receptor and activation of structural gene products may be responsible for such activity. Alternatively, exogenous compounds may interfere with the degradation of existing proteins and favor de novo protein synthesis in insects^60^. TPP, DEM, and PBO work synergistically with eugenol. More specifically, PBO can be used to enhance the toxicity of eugenol towards the larvae of *A. aegypti*.

## Conclusion

Eugenol caused dose–response mortality in larvae, and the effect on mortality did not change for 30 generations. Despite variations in LC50 across generations, the mosquitoes' susceptibility to eugenol did not appear to vary in terms of median lethal concentration. The LC50 value initially decreased till F9 generation and then rise to the original LC50 value after F24 generation and remained almost stable till F30. The activity of metabolic detoxifying enzymes followed a similar pattern showing initial decrease for up to F4–F10 generations and then gradual increase in subsequent generations. Thus, there is a relationship between higher toxicity and lower detoxifying enzyme activity in early generations and a subsequent reduction of toxicity with increasing detoxifying enzyme activity in later generations. In cytochrome p450, the pattern of an initial decrease and subsequent increase in enzyme activity was observed in the studied generation. The pattern matches with the initial increase in toxicity and later decrease in toxicity of eugenol on exposed mosquito populations. Hence, there might be a relationship between cytochrome p450 enzyme activity and the susceptibility of the larvae to eugenol. However, no such prominent pattern was observed for esterases and GST activities with respect to eugenol toxicity. GST remained below the control group throughout the generation and esterase increased significantly than control after the passage of 5–10 generations. When PBO was used as a synergist, the toxicity of eugenol increased significantly. Combining PBO with eugenol affected cytochrome P450 as well as esterases. P450 activity was observed to decline significantly by almost threefold. Overall, the authors suggest that eugenol would effectively work as an *A. aegypti* larvicide for a longer duration, without impairing its effectiveness. Combining synergists with eugenol would increase the toxicity of eugenol on *A. aegypti* larvae.

## Supplementary Information

Supplementary Information.
