# Supplementary material for: Persistent susceptibility of Aedes aegypti to eugenol
Source: Sci Rep. 2022 Feb 10;12:2277. doi: 10.1038/s41598-022-06302-8 (PMC8831528; doi:10.1038/s41598-022-06302-8)
Supplement: Supplementary file 1 — Supplementary Information. [file 41598_2022_6302_MOESM1_ESM.docx]

**Persistent susceptibility of *Aedes aegypti* to eugenol**

Kamal Adhikari^1^, Bulbuli Khanikor^2^*, Riju Sarma^3^

1&3 Research scholar, Department of Zoology, Gauhati University, Guwahati, Assam, India

Email id- [kamaladhikari246@gmail.com](mailto:kamaladhikari246@gmail.com) (KA)

Email id- [sarmariju64@gmail.com](mailto:sarmariju64@gmail.com) (RS)

2 Assistant professor, Department of Zoology, Gauhati University, Guwahati, Assam, India

Correspondence to: Dr Bulbuli Khanikor

*Corresponding author- Email: [khanikorbulbuli@yahoo.co.in](mailto:khanikorbulbuli@yahoo.co.in)

Supplementary Table 1. Percent mortality of *Aedes aegypti* 4th instar larvae in response to different concentrations of eugenol in different generations (%Mortality±SE).

| Con Gen | 1ppm | 5ppm | 10ppm | 25ppm | 50ppm | 100ppm | 250ppm | 500ppm |
| --- | --- | --- | --- | --- | --- | --- | --- | --- |
| F0 | 0±0 | 1.3±0.33 | 8±0.33 | 12±1.83 | 30.67±2.83 | 64+2.83 | 98.66±0.33 | 100±0 |
| F1 | 0±0 | 1.3±0.33 | 6.67±0.33 | 13.33±0.83 | 32±1.22 | 62.67±1.22 | 98.66±0.33 | 100±0 |
| F2 | 0±0 | 0±0 | 8±0.33 | 21.33±0.58 | 32±0.83 | 66.67±1.79 | 100±0 | 100±0 |
| F3 | 1.3±0.33 | 2.67±0.33 | 8±0.58 | 25.33±0.89 | 36±1 | 58.67±1.79 | 100±0 | 100±0 |
| F4 | 0±0 | 5.3±0.33 | 10.67±0.89 | 22.67±0.33 | 41.33±1 | 69.33± 1.22 | 100±0 | 100±0 |
| F5 | 0±0 | 1.3±0.33 | 10.67±0.89 | 26.67±0.89 | 38.67±0.59 | 65.33±1.22 | 100±0 | 100±0 |
| F6 | 0±0 | 2.67±0.33 | 16±0.89 | 28±0.59 | 41.33±0.89 | 65.33±1.83 | 98.66±0.33 | 100±0 |
| F7 | 0±0 | 4±0 | 9.33±1.22 | 26.67±0.59 | 44±0.89 | 76±0.89 | 96±0.58 | 100±0 |
| F8 | 0±0 | 1.3±0.33 | 10.67±0.33 | 33.33±0.83 | 37.33±1.22 | 81.33±1.22 | 100±0 | 100±0 |
| F9 | 0±0 | 1.3±0.33 | 10.67±0.59 | 33.33±0.67 | 40±2.83 | 82.67±0.89 | 100±0 | 100±0 |
| F10 | 0±0 | 4±0 | 10.67±0.89 | 32±1.83 | 41.33±1.83 | 80±2.83 | 100±0 | 100±0 |
| F11 | 0±0 | 2.67±0.33 | 9.33±0.89 | 28±0.83 | 41.33±1 | 90.67±0.67 | 96±0.58 | 100±0 |
| F12 | 0±0 | 1.3±0.33 | 9.33±1 | 28±1.22 | 40±0.59 | 73.33±2.83 | 100±0 | 100±0 |
| F13 | 0±0 | 2.67±0.33 | 10.67±1 | 26.67±0.33 | 37.33±0.83 | 70.67±1.83 | 100±0 | 100±0 |
| F14 | 0±0 | 2.67±0.33 | 13.33±0.83 | 24±0.59 | 37.33±0.89 | 72±0.89 | 98.66±0.33 | 100±0 |
| F15 | 0±0 | 4±0.89 | 9.33±1.22 | 25.33±1.22 | 38.67±0.59 | 65.33±1.83 | 98.66±0.33 | 100±0 |
| F16 | 0±0 | 1.33±0.33 | 13.33±0.83 | 24±0.59 | 38.67±1.83 | 65.33±0.83 | 96±0.58 | 100±0 |
| F17 | 0±0 | 1.33±0.33 | 13.33±0.83 | 24±0.89 | 40±0.67 | 61.33±0.89 | 96±0.58 | 100±0 |
| F18 | 0±0 | 1.33±0.33 | 10.67±0.83 | 22.67±0.83 | 38.67±1.83 | 61.33±0.89 | 98.66±0.33 | 100±0 |
| F19 | 0±0 | 1.33±0.33 | 9.33±.033 | 22.67±1 | 34.66±1.83 | 57.33±0.89 | 100±0 | 100±0 |
| F20 | 0±0 | 4±0 | 8±89 | 21.33±0.89 | 34.66±0.59 | 56±1.22 | 100±0 | 100±0 |
| F21 | 0±0 | 1.33±0.33 | 10.66±0.1.22 | 21.33±0.83 | 33.33±1.22 | 56±0.89 | 100±0 | 100±0 |
| F22 | 0±0 | 1.33±0.33 | 9.33±1.22 | 17.33±0.33 | 34.66±1.22 | 57.33±0.89 | 98.66±0.33 | 100±0 |
| F23 | 0±0 | 1.33±0.33 | 9.33±0.83 | 17.33±0.83 | 33.33±1.22 | 57.33±2.83 | 97.33±0.33 | 100±0 |
| F24 | 0±0 | 2.66±0.33 | 9.33±1 | 20±0 | 32±0.59 | 54.66±1.83 | 97.33±0.33 | 100±0 |
| F25 | 0±0 | 2.66±0.33 | 9.33±1.22 | 20±0.59 | 32±1.22 | 53.33±0.59 | 97.33±0.33 | 100±0 |
| F26 | 0±0 | 2.66±0.33 | 9.33±0.89 | 20±1.83 | 32±2.83 | 54.66±1.22 | 97.33±0.33 | 100±0 |
| F27 | 0±0 | 2.66±0.33 | 9.33±0.83 | 20±0.89 | 32±2.89 | 54.66±1.22 | 97.33±0.33 | 100±0 |
| F28 | 0±0 | 2.66±0.33 | 9.33±0.83 | 18.66±0.83 | 30.66±0.83 | 54.66±1.22 | 97.33±0.33 | 100±0 |
| F29 | 0±0 | 2.66±0.33 | 10.66±0.33 | 18.66±0.83 | 29.33±0.83 | 56±1 | 96±0 | 100±0 |
| F30 | 0±0 | 1.33±0.33 | 10.66±0.33 | 17.33±2.83 | 29.33±1.22 | 54.66±2.83 | 96±0 | 100±0 |

Supplementary Table 2. LC50 values of Eugenol in successive generations of *Aedes aegypti*

| Generations | LC50 value  (ppm) | Regression equation | 95% confidence limit | | Chi- square value |
| --- | --- | --- | --- | --- | --- |
|  |  |  | Lower limit | Upper limit |  |
| F0 | \| 63.48 \| \| --- \| | Y=0.67+2.40X | 55.327 | 74.053 | 30.182 |
| F1 | 62.09 | Y=0.77+2.36X | 54.636 | 73.116 | 28.161 |
| F2 | 59.34 | Y=0.79+2.37X | 49.553 | 66.169 | 22.169 |
| F3 | 55.35 | Y=1.22+2.15X | 26.003 | 85.291 | 213.67 |
| F4 | 52.10 | Y=1.27+2.17X | 42.736 | 59.079 | 16.379 |
| F5 | 50.96 | Y=1.10+2.28X | 43.222 | 58.690 | 19.337 |
| F6 | 50.33 | Y=1.31+2.16X | 40.569 | 56.155 | 20.458 |
| F7 | 48.28 | Y=1.21+2.25X | 39.900 | 54.575 | 8.961 |
| F8 | 45.76 | Y=1.13+2.32X | 38.588 | 52.243 | 18.668 |
| F9 | 43.67 | Y=1.08+2.40X | 36.881 | 49.882 | 16.564 |
| F10 | 43.79 | Y=1.17+2.33X | 36.232 | 49.184 | 14.655 |
| F11 | 47.99 | Y=1.15+2.29X | 40.179 | 54.735 | 11.617 |
| F12 | 48.38 | Y=1.04+2.34X | 41.425 | 55.952 | 16.050 |
| F13 | 49.98 | Y=1.13+2.28X | 42.088 | 57.224 | 17.852 |
| F14 | 51.2 | Y=1.21+2.21X | 42.443 | 58.203 | 16.770 |
| F15 | 53.13 | Y=1.19+2.21X | 44.139 | 60.537 | 21.115 |
| F16 | 53.91 | Y=1.18+2.20X | 44.802 | 61.450 | 17.977 |
| F17 | 54.89 | Y=1.21+2.18X | 45.682 | 62.845 | 19.062 |
| F18 | 55.68 | Y=1.10+2.23X | 47.206 | 64.337 | 19.042 |
| F19 | 57.79 | Y=1.27+2.12X | 34.313 | 94.663 | 150.44 |
| F20 | 59.07 | Y=1.2+2.19X | 50.052 | 68.613 | 25.184 |
| F21 | 59.86 | Y=1.05+2.22X | 50.808 | 69.288 | 28.528 |
| F22 | 60.87 | Y=1.00+2.23X | 52.645 | 71.460 | 25.420 |
| F23 | 62.47 | Y=1.01+2.21X | 53.753 | 73.153 | 23.277 |
| F24 | 63.00 | Y=1.11+2.15X | 53.372 | 73.360 | 24.438 |
| F25 | 63.12 | Y=1.140+2.14X | 53.890 | 74.142 | 24.969 |
| F26 | 63.30 | Y=1.087+2.171X | 53.372 | 73.360 | 25.014 |
| F27 | 63.30 | Y=1.087+2.171X | 53.372 | 73.360 | 25.014 |
| F28 | 63.90 | Y=1.0936+2.16X | 54.412 | 74.721 | 25.905 |
| F29 | 64.24 | Y=1.1292+2.141X | 54.358 | 75.001 | 24.719 |
| F30 | 64.50 | Y=1.063+2.1752 | 56.227 | 77.178 | 27.952 |

Supplementary Table 3: GST, CYT P450 and Esterase activities in 4th instar larva of successive generations of *Aedes aegypti* in response to respective LC50 dose of Eugenol

| Gen | GST activity (control) | GST activity (eugenol treated) | CYT P450 ACTIVITY (control) | CYT P450 activity (eugenol) | α- Esterase activity (control) | α- Esterase activity (eugenol) | β- Esterase activity (control) | β- esterase activity (eugenol) |
| --- | --- | --- | --- | --- | --- | --- | --- | --- |
| F0 | 0.51 | 0.47 | 4.20×10^-6^ | 3.92×10^-6^ | 2.14×10^-6^ | 1.59×10^-6^ | 2.42×10^-6^ | 2.54×10^-6^ |
| F1 | 0.50 | 0.39 | 4.21×10^-6^ | 4.81×10^-6^ | 2.13×10^-6^ | 1.63×10^-6^ | 2.41×10^-6^ | 2.54×10^-6^ |
| F2 | 0.52 | 0.25 | 4.20×10^-6^ | 4.09×10^-6^ | 2.12×10^-6^ | 1.66×10^-6^ | 2.39×10^-6^ | 1.48×10^-6^ |
| F3 | 0.51 | 0.22 | 4.22×10^-6^ | 4.13×10^-6^ | 2.14×10^-6^ | 1.27×10^-6^ | 2.41×10^-6^ | 2.26×10^-6^ |
| F4 | 0.50 | 0.19 | 4.22×10^-6^ | 4.13×10^-6^ | 2.14×10^-6^ | 1.42×10^-6^ | 2.42×10^-6^ | 1.8×10^-6^ |
| F5 | 0.50 | 0.13 | 4.20×10^-6^ | 3.99×10^-6^ | 2.15×10^-6^ | 2.11×10^-6^ | 2.39×10^-6^ | 2.42×10^-6^ |
| F6 | 0.50 | 0.20 | 4.20×10^-6^ | 3.87×10^-6^ | 2.15×10^-6^ | 2.42×10^-6^ | 2.40×10^-6^ | 2.34×10^-6^ |
| F7 | 0.52 | 0.21 | 4.22×10^-6^ | 3.73×10^-6^ | 2.14×10^-6^ | 2.63×10^-6^ | 2.40×10^-6^ | 2.81×10^-6^ |
| F8 | 0.51 | 0.22 | 4.22×10^-6^ | 3.22×10^-6^ | 2.13×10^-6^ | 2.55×10^-6^ | 2.39×10^-6^ | 2.97×10^-6^ |
| F9 | 0.50 | 0.20 | 4.22×10^-6^ | 3.52×10^-6^ | 2.14×10^-6^ | 2.70×10^-6^ | 2.39×10^-6^ | 2.96×10^-6^ |
| F10 | 0.50 | 0.22 | 4.20×10^-6^ | 3.58×10^-6^ | 2.14×10^-6^ | 2.74×10^-6^ | 2.40×10^-6^ | 3.04×10^-6^ |
| F11 | 0.51 | 0.25 | 4.20×10^-6^ | 3.68×10^-6^ | 2.14×10^-6^ | 2.72×10^-6^ | 2.41×10^-6^ | 3.13×10^-6^ |
| F12 | 0.50 | 0.27 | 4.20×10^-6^ | 3.82×10^-6^ | 2.15×10^-6^ | 2.87×10^-6^ | 2.42×10^-6^ | 2.94×10^-6^ |
| F13 | 0.50 | 0.30 | 4.20×10^-6^ | 3.95×10^-6^ | 2.14×10^-6^ | 2.92×10^-6^ | 2.41×10^-6^ | 3.21×10^-6^ |
| F14 | 0.50 | 0.32 | 4.20×10^-6^ | 4.25×10^-6^ | 2.14×10^-6^ | 2.98×10^-6^ | 2.43×10^-6^ | 3.18×10^-6^ |
| F15 | 0.51 | 0.34 | 4.22×10^-6^ | 4.42×10^-6^ | 2.15×10^-6^ | 3×10^-6^ | 2.42×10^-6^ | 3.19×10^-6^ |
| F16 | 0.51 | 0.35 | 4.22×10^-6^ | 4.42×10^-6^ | 2.13×10^-6^ | 3.04×10^-6^ | 2.42×10^-6^ | 3.29×10^-6^ |
| F17 | 0.51 | 0.35 | 4.22×10^-6^ | 4.46×10^-6^ | 2.13×10^-6^ | 3.12×10^-6^ | 2.42×10^-6^ | 3.32×10^-6^ |
| F18 | 0.50 | 0.36 | 4.21×10^-6^ | 4.51×10^-6^ | 2.14×10^-6^ | 3.21×10^-6^ | 2.41×10^-6^ | 3.37×10^-6^ |
| F19 | 0.50 | 0.358 | 4.20×10^-6^ | 4.95×10^-6^ | 2.14×10^-6^ | 3.36×10^-6^ | 2.41×10^-6^ | 3.57×10^-6^ |
| F20 | 0.50 | 0.3612 | 4.21×10^-6^ | 5.09×10^-6^ | 2.15×10^-6^ | 3.38×10^-6^ | 2.41×10^-6^ | 3.62×10^-6^ |
| F21 | 0.51 | 0.395 | 4.20×10^-6^ | 5.49×10^-6^ | 2.15×10^-6^ | 3.53×10^-6^ | 2.42×10^-6^ | 3.80×10^-6^ |
| F22 | 0.50 | 0.4068 | 4.20×10^-6^ | 5.25×10^-6^ | 2.14×10^-6^ | 3.54×10^-6^ | 2.42×10^-6^ | 3.65×10^-6^ |
| F23 | 0.49 | 0.40367 | 4.21×10^-6^ | 5.38×10^-6^ | 2.13×10^-6^ | 3.40×10^-6^ | 2.41×10^-6^ | 3.51×10^-6^ |
| F24 | 0.49 | 0.3875 | 4.21×10^-6^ | 5.57×10^-6^ | 2.13×10^-6^ | 3.56×10^-6^ | 2.41×10^-6^ | 3.67×10^-6^ |
| F25 | 0.50 | 0.4215 | 4.20×10^-6^ | 5.44×10^-6^ | 2.14×10^-6^ | 3.41×10^-6^ | 2.40×10^-6^ | 3.69×10^-6^ |
| F26 | 0.50 | 0.425 | 4.22×10-6 | 5.46×10^-6^ | 2.14×10^-6^ | 3.56×10^-6^ | 2.42×10^-6^ | 3.85×10^-6^ |
| F27 | 0.51 | 0.4319 | 4.22×10-6 | 5.44×10^-6^ | 2.14×10^-6^ | 3.65×10^-6^ | 2.43×10^-6^ | 3.94×10^-6^ |
| F28 | 0.50 | 0.441 | 4.21×10-6 | 5.55×10^-6^ | 2.13×10^-6^ | 3.56×10^-6^ | 2.42×10^-6^ | 3.94×10^-6^ |
| F29 | 0.50 | 0.441 | 4.2×10-6 | 5.57×10^-6^ | 2.13×10^-6^ | 3.56×10^-6^ | 2.42×10^-6^ | 3.94×10^-6^ |
| F30 | 0.51 | 0.447 | 4.2×10-6 | 5.62×10^-6^ | 2.14×10^-6^ | 3.59×10^-6^ | 2.42×10^-6^ | 3.92×10^-6^ |

Supplementary Table 4. Effect of synergists used with eugenol on the LC50 values of F30 generation.

| Generations | LC50 value  (ppm) | Regression equation | 95% confidence limit | | Chi- square value |
| --- | --- | --- | --- | --- | --- |
|  |  |  | Lower limit | Upper limit |  |
| Eugenol alone | 64.5 | Y=1.049+2.169X | 56.227 | 77.178 | 26.087 |
| Eugenol+ PBO | 49.98 | Y=1.12638+2.28025X | 42.088 | 57.224 | 17.852 |
| Eugenol + TPP | 57.79 | Y=1.27980+2.11146X | 34.313 | 94.663 | 19.042 |
| EUGENOL + DEM | 63 | Y=1.11433+2.15886X | 53.372 | 73.360 | 24.438 |

Supplementary Table 5. Effect of synergists used with eugenol on the detoxification enzyme of *Aedes aegypti* at the F30 generation.

| Treatment | GST (conjugate produced/min/mg protein) | Cyt P450 (P450/min/mg protein) | α- Esterase  (μM of product formed/min/mg protein) | β- Esterase  (μM of product formed/min/mg protein) |
| --- | --- | --- | --- | --- |
| Eugenol alone | 0.447 | 5.62×10^-6^ | 3.59×10^-6^ | 3.92×10^-6^ |
| Eugenol +PBO | 0.447 | 2.17×10^-6^ | 2.63×10^-6^ | 2.97×10^-6^ |
| Eugenol + TPP | 0.447 | 5.62×10^-6^ | 1.27×10^-6^ | 1.8×10^-6^ |
| Eugenol + DEM | 0.300 | 5.62×10^-6^ | 3.59×10^-6^ | 3.92×10^-6^ |
